# How can we improve patients’ access to new drugs under uncertainties? : South Korea’s experience with risk sharing arrangements

**DOI:** 10.1186/s12913-021-06919-x

**Published:** 2021-09-14

**Authors:** Boram Lee, Eun-Young Bae, SeungJin Bae, Hyun-Jin Choi, Kyung-Bok Son, Young-Sil Lee, Suhyun Jang, Tae-Jin Lee

**Affiliations:** 1 Department of Public Health Science, Graduate School of Public Health, Seoul National University, Seoul, South Korea; 2 College of Pharmacy, Gyeongsang National University, Jinju, South Korea; 3 College of Pharmacy, Ewha Womans University, Seoul, South Korea; 4 College of Pharmacy, Gachon University, Incheon, South Korea; 5 Institute of Pharmaceutical Sciences, Gachon University, Incheon, South Korea; 6 Institute of Health and Environment, Seoul National University, Seoul, South Korea

**Keywords:** Cost-effectiveness, Drug reimbursement, Economic evaluation, Access to new drugs, Risk sharing arrangement, South Korea

## Abstract

**Background:**

New drugs including cancer drugs and orphan drugs are becoming increasingly more expensive. Risk sharing arrangements (RSAs) could manage the risk based on both financial impact and the health outcome of new drugs if reimbursed. To improve patients’ access to new drugs under uncertainties, many developed countries have adopted RSAs. In this study, we aimed to understand the effects of RSAs in South Korea on patients’ access.

**Methods:**

We reviewed current status of RSA drugs in South Korea. The number of appraisals and time gap between market approval and reimbursement per RSA drug were considered to quantify improvement of patients’ access as they showed how rapidly decisions on reimbursement of RSA drugs were derived. Then, we applied a comparative analysis to determine whether the RSA drugs in South Korea were reimbursed in the UK, Italy, and Australia. Most data for this study were obtained from websites of the governmental department/agencies responsible for appraisal of drug reimbursement in each country. And literatures related to RSAs were investigated as well.

**Results:**

The eligibility for Korean RSAs had two key components - drugs for cancer and rare diseases and not having other alternative treatments. As of the first half of 2019, there were 39 RSA drugs reimbursed in South Korea, the majority of which were financial-based schemes. Refund and expenditure cap were the representative types (89.7%). After introduction of RSAs, the time gap and number of appraisals were decreased. Based on the indications of RSA drugs, the level of drug coverage in South Korea was found lower than Italy, similar to the UK, and higher than Australia.

**Conclusions:**

RSAs in South Korea significantly enhanced patients’ access to new drugs and led to the alleviation of patients’ out-of-pocket expenses. The drug coverage of South Korea had a level comparable to that of other countries. This study provides implications for countries that have a dual mission of containing pharmaceutical expenditure and improving access to new drugs.

**Supplementary Information:**

The online version contains supplementary material available at 10.1186/s12913-021-06919-x.

## Background

New drugs, especially cancer drugs and orphan drugs, are becoming increasingly more expensive. Unfortunately, however, a higher price does not always guarantee greater effectiveness. Nevertheless, most patients prefer early access to new drugs. However, payers and patients cannot easily afford them. This problem can be found in many countries, leaving health authorities with a challenging task of balancing between two issues – early access and cost containment.

Since the early 2000s, risk sharing arrangements (RSAs) have been introduced to manage the “risk” based on both financial impact and the health outcome of new drugs [1, 2]. Payers and manufacturers share the burden of these risks to achieve a common goal: expansion of coverage. There are three categories of risk sharing schemes: (1) performance-based schemes, which consider clinical efficacy, with the outcome of patients linked to price and/or coverage of drugs; (2) financial-based schemes, which are related to the cost of drugs, such as price discount, rebate, price-volume agreements, and expenditure/utilization cap; and (3) evidence-generating schemes, which are implemented to collect more sufficient evidence in the real world.

Decisions of drug reimbursement may be deferred due to a lack of information, ultimately leading to uncertainties regarding these new drugs [3]. This deferment can be disappointing for patients who desire new drugs. However, the authorities must allocate healthcare funding over the entire population based on clinical efficacy and cost-effectiveness. Under this circumstance, RSAs can be a notable policy tool for satisfying different stakeholders. Patients receive timely access while payers (also called as insurers, governments, or purchasers) reduce a financial burden and an uncertainty of evidence. Further, pharmaceutical companies can improve market access and easily adhere to the global pricing strategy [4].

Over two-thirds of the Organization for Economic Co-operation and Development and European Union countries have been utilizing or had utilized RSAs by 2019 [5]. Goals, schemes, and implementations for risk sharing differ according to the background of each country. There are several other terms on RSAs—managed entry agreements, special pricing arrangements, patient access schemes, managed access schemes, and so on. Nonetheless, their initiatives begin at the same line – scarce resources in healthcare. Ferrario and Kanavos [6] suggested three objectives for these arrangements: managing budgetary impact, purchasing cost-effective therapeutics, and monitoring utilization.

South Korea also introduced RSAs to improve patient’s access to new drugs in 2013. Since December 2006, South Korea has implemented a positive listing system for drug reimbursement, in which pharmaceutical companies should submit evidence of cost-effectiveness for most drugs. However, high-price drugs for cancer and rare diseases often miss these reimbursements due to failure to prove cost-effectiveness. As the number of these cases increased, calls from patients to improve the access to those drugs continued to increase, to which the Korean government responded by introducing RSAs.

The main purpose of this study is to describe the features and implications of RSAs in South Korea and review their effects on patients’ access to new drugs. Additionally, we aim to compare the levels of patients’ access to new drugs between South Korea and several developed countries – the UK, Australia and Italy. This may yield some implications for other countries struggling between increasing pharmaceutical expenditure and the need for early access.

### Risk sharing arrangements in South Korea

In September 2013, the Ministry of Health and Welfare (MoHW) in South Korea officially announced the introduction of RSAs to help alleviate the financial burden associated with the treatment of patients with severe diseases and allow better access to new drugs. RSAs were restricted to the implementation of drugs that could be hardly accessed otherwise. To be eligible for RSAs, a drug should meet the following three criteria: (1) it must be a cancer drug or orphan drug for rare diseases; (2) there should be neither alternative treatments nor equivalent therapeutic drugs available; and (3) it should be used to treat serious, life-threatening condition. Alternatively, a drug can be eligible if the Drug Reimbursement Evaluation Committee (DREC) reaches an agreement based on the severity of the disease, social impacts, influence on public health, and other factors. [7]. The period of this contract can be up to 4 years; however, for an extension to be granted, a re-evaluation is necessary.

There are four stipulated types of RSAs in South Korea: 1) conditional treatment continuation and money back guarantee, a type that is based on individual patient’s response after treatment. If the response meets the predefined treatment goal, the drug continues to be reimbursed by the payer, the National Health Insurance Service (NHIS). Otherwise, the company should refund the full drug costs to the NHIS; 2) expenditure cap, which sets the total annual expenditure of the drug in advance. The company pays back an agreed rate of the exceeding amount to the NHIS; 3) a refund type where the company refunds a certain percentage of the nominal price of the drug to the NHIS; 4) utilization cap or fixed cost per patient, which designates the upper limit of utilization of the drug per patient. Further, the company covers the cost of the drug beyond pre-agreed utilization. Other ad hoc types of RSAs can also be suggested by the company [8].

Since the introduction of RSAs in 2013, the Korean government has allowed this agreement based on product alone but not indication. Accordingly, only one agreement was allowed for each RSA drug despite its indications being two or more. Since October 2016, however, adding new indications to the existing contract of RSA drugs has become possible. As a result, the new indication had to be appraised by the DREC, which can amend the terms of the contract through a negotiation between the NHIS and a pharmaceutical company for the remaining contract period. All RSA drugs should be monitored for their utilization at specific intervals. At the end of the period, re-evaluation of the eligibility for extension of the contract should be performed. If there is a therapeutic alternative to a specific RSA drug, the contract cannot be extended. If its generic is listed during the contract period, the contract must be immediately terminated.

Despite these approaches through RSAs to expand the coverage of the Korean national health insurance (NHI), some drugs are still not reimbursed by the NHIS. In the case of ultra-rare diseases, it appeared almost impossible to generate evidence for cost-effectiveness. In December 2014, the MoHW introduced an exemption of economic evaluation (EEE) policy and implemented it with very strict criteria. Only cancer drugs or orphan drugs could receive a waiver of economic evaluation when they satisfy three requirements: (1) the condition is so severe that patients’ lives are threatened and there is no alternative intervention; (2) the number of patients is too small to generate evidence; and (3) the drug is reimbursed in at least three of the following seven countries: the UK, Italy, France, Germany, Switzerland, the US, and Japan [9]. Moreover, since September 2016, every new drug introduced by the EEE policy should share its risk in the form of an expenditure cap.

### Risk sharing arrangements in the UK, Australia, and Italy

National health service (NHS) England has patient access schemes (PAS) to manage the risk of uncertainty and cancer drugs fund (CDF) to provide patients with faster access to new cancer treatments [10, 11]. Tracing back to its origin in 2002, there was an agreement between the Department of Health (DoH) and pharmaceutical companies on the long-term cost-effectiveness of multiple sclerosis patients [12]. In 2007, the DoH and the National Institute for Health and Care Excellence (NICE) proposed a legislation of PAS which was applied in the context of value-based pricing under a pharmaceutical price regulation scheme [13]. There are two broad categories in PAS: a simple discount scheme and a complex scheme [10]. The simple discount scheme involves discounting the price of a drug while complex schemes involve all other types including rebates, stock supplied at zero cost, dose capping and performance-based risk sharing. The patient access schemes liaison unit reviews the PAS proposals submitted by pharmaceutical companies, which are presented at the consultation with the NICE. To contain drug cost, simple discount was the most frequent type of PAS [14, 15]. In April 2011, the CDF was established, as a temporary measure until March 2016 [11], to provide early access to cancer drugs not recommended by the NICE. However, before long, the NHS faced financial pressure and CDF was completely amended in July 2016. As a key part of CDF, CDF-managed access agreement is considered to collect data. This contributes to the management of clinical uncertainties related to the NICE appraisal [16]. Thus, the current new CDF operates under population-level coverage with an evidence development (CED) scheme [5].

In 1998, the Department of Health of Australia reached an agreement with a pharmaceutical company to minimize the risks related to appropriateness and cost-effectiveness of drugs reimbursed by the Pharmaceutical Benefits Scheme (PBS) [17]; this served as the first agreement. The deeds of agreement was then introduced in 2003 to cover the two types of arrangements: special pricing arrangement (SPA) and risk sharing arrangement (RSA). SPA has a dual price — published price and real, confidential price. The refund by the companies should follow according to the difference between these two prices. RSA addresses various risks derived from the reimbursement of a new drug. Cost-effectiveness, overall cost to the PBS, overall health gain requiring data collection and monitoring, and overall utilization related to the number of patients are representative risks handled via RSA [18]. These two arrangements mainly manage financial-related risks and are sometimes used in combination schemes, refund with subsidization or expenditure cap [4]. Since 2011, the Australian PBS has operated managed entry schemes (MES) to share the risks associated with uncertainty for efficacy and help patients receive earlier access to drugs [19]. New drugs with high clinical needs are eligible for MES. Under MES, an initial price of the drug is established and evidence from clinical trials must be submitted to the Pharmaceutical Benefits Advisory Committee (PBAC) within a specified timeframe. By reviewing resubmitted data, the PBAC can propose a final recommendation for the PBS listing and the drug price will be reset at this future time. Although not a form of risk sharing, the life-saving drugs program (LSDP) has helped Australian patients access drugs for life threatening and rare diseases since 1995 [20].

Italian NHS (Servizio Sanitario Nazionale, SSN) named its risk sharing policy managed entry agreement (MEA) for cost containment and patients’ accessibility. MEA provides its application via two schemes—performance-based and financial-based [21]. In July 2006, the Agenzia Italiana del Farmaco (AIFA) agreed on its first MEA. Italian MEAs are classified into four types. Payment by result and risk sharing are classified as performance-based schemes while cost sharing and capping are classified as financial-based. If patients do not respond to treatment, risk sharing model proposes a discount to treatment cost. However, under payment by result model, the pharmaceutical company should offer 100% payback. Cost sharing model discounts treatment cost of medication while capping forces the expenditure limit. Table 1 shows a comparative summary of policies in these countries.

**Table 1:** RSA and related policies in the UK, Italy, Australia, and South Korea

	South Korea	UK	Italy	Australia
*main policy for improving access to new drugs*				
policy name	risk sharing arrangements (RSA)	patient access schemes (PAS)	managed entry agreement (MEA)	managed entry schemes (MES)
responsible department/agency	Health Insurance Review and Assessment Service (HIRA)	National Institute for Health and Care Excellence (NICE)	Agenzia Italiana del Farmaco (AIFA)	Pharmaceutical Benefits Scheme (PBS)
year of introduction	2013	2007	2006	2003 (2011^a^)
target drugs to be applied	∙ cancer drugs or orphan drugs ∙ no alternative treatments or drugs ∙ used in serious, life-threatening condition	no limitation	no limitation	no limitation
types of risk sharing	∙ CTC and money back guarantee ∙ expenditure cap ∙ refund ∙ utilization cap or fixed cost per patient ∙ others can be suggested	∙ simple discount scheme ∙ complex scheme	∙ payment by result (PbR) ∙ risk sharing (RS) ∙ cost sharing (CS) ∙ capping	∙ special pricing arrangement (SPA) ∙ risk sharing arrangement (RSA)
*related policies*				
policy name	exemption of economic evaluation (EEE)	cancer drugs fund (CDF)		life-saving drugs program (LSDP)
year of introduction	2014	2011		1995
target drugs to be applied	∙ used in serious, life-threatening condition and no alternative intervention ∙ the number of patients is too small to generate evidence ∙ reimbursed in at least three of the seven countries^b^	cancer drugs		for life threatening and rare diseases

^a^The deeds of agreement was introduced in 2003 and the Australian Pharmaceutical Benefits Scheme (PBS) has operated managed entry schemes since 2011

^b^the UK, Italy, France, Germany, Switzerland, the US, and Japan

*CTC* conditional treatment continuation

## Methods

We described the current status and history of RSA drugs in South Korea including their indications, type of risk sharing, and changes of reimbursement condition. To understand its effect on patients’ access to drugs, we focused on how rapidly decisions on reimbursement of RSA drugs were derived, using two measures. One is the time gap between the date of market approval by the Ministry of Food and Drug Safety (MFDS) and the first date of reimbursement by the NHIS. The other is the number of appraisals per RSA drug completed by the DREC before it reaches a contract for RSA.

Subsequently, we sought to determine whether the RSA drugs in South Korea are reimbursed in other countries such as the UK, Italy, and Australia. Based on their indications, each RSA drug in South Korea was investigated to determine whether and how they are reimbursed – through conventional reimbursement, RSAs, or other special programs; this could help us to understand patients’ access to those drugs in South Korea. The UK (NHS England), Italy, and Australia were selected as they are very active at adopting RSAs and have either national health service or tax-funded universal health insurance. Likewise, South Korea has NHI that only has a single payer, the NHIS; it covers all Koreans as the same beneficiaries. In this context, circumstances surrounding decision making in drug reimbursement seem to be quite similar among these four countries. Furthermore, there are some advantages to obtaining official data among the selected countries.

For the Korean data, we searched the official governmental documents online. Official websites of Health Insurance Review and Assessment Service (HIRA) that operates the DREC, NHIS, MFDS, and MoHW were the main sources of the Korean data. We reviewed regulations for reimbursement, proceedings/minutes of the appraisal by the DREC, and a drug list of reimbursement by the NHIS [7–9, 22–25]. In addition, literatures related to RSAs in South Korea were investigated, such as peer-reviewed articles and nonconventional publications including government reports, press releases, and dissertations/theses. For the data from the UK, Italy, and Australia, websites for the governmental department/agencies responsible for appraisal of drug reimbursement in each country were included: NHS England and the NICE in the UK, AIFA in Italy, and the PBS in Australia. We reviewed the lists of PAS, CDF, and national tariff excluded drugs list for the UK data [10, 26–28]. AIFA discloses the reimbursement list for class H, class A drugs, and MEAs [29–31]. We performed a more comprehensive review for the Australian PBS data. To obtain information regarding MES and RSA, we examined every public summary document that mentioned MES and financial management. We identified the outcomes for which the PBAC was only recommended while the rejected or deferred results were excluded [32]. The pharmaceutical schedules were checked for drugs subsidized under the PBS and other arrangements, especially SPA [33–35]. Our last update was performed in July 2019.

## Results

**Fig. 1:**
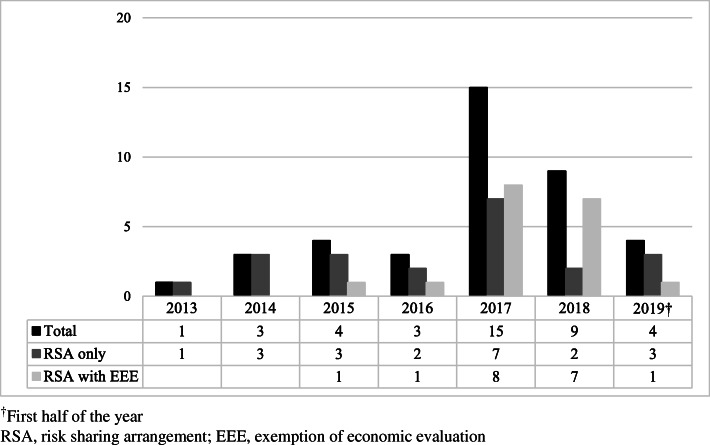
Number of newly contracted RSA drugs per year

Figure 1 shows the number of newly contracted RSA drugs in South Korea per year. As of the first half of 2019, 39 individual drugs were or had been reimbursed under RSAs. RSAs with EEE were applied to 18 drugs. In 2017, the largest number of drugs (i.e., 15 drugs) was reimbursed under RSAs. Over three quarters of the total were cancer drugs (30 drugs, 76.9%).

The current status and characteristics of the RSA drugs are described in Table 2. Further, these drugs were categorized according to their active or expired RSA contracts. Although the activation of 6 drugs was expired, they are still reimbursed. Financial-based schemes were most predominant in Korean RSAs, with one exception, clofarabine, the very first RSA drug under evidence-generating scheme. The company suggested CED type of risk sharing, where clofarabine was covered for all the relevant patients. The most common financial schemes were refund (17 drugs, 43.6%) and expenditure cap (23 drugs, 59.0%). All drugs under the expenditure cap were exempted from economic evaluation despite two having their arrangements settled before September 2016. Five drugs (nivolumab, pembrolizumab, cabozantinib, daratumumab, and nusinersen) had a combined scheme with refund and expenditure cap. Utilization cap, time cap, and discounted treatment initiation type of risk sharing also existed for each drug.

**Table 2:** List of RSA drugs in South Korea

Brand name	Active substance	Type	Start date (End date)	Note	
*Activation ongoing*					
Erbitux	cetuximab	refund	5-Mar-2014	re-evaluation for RSA (1-Jul-2018)	
Xtandi	enzalutamide	refund	1-Nov-2014	re-evaluation for RSA (13-Feb-2019)	
Soliris	eculizumab	refund	1-Oct-2015	refund pilot program (1-Oct-2012), Essential drug	
Caprelsa	vandetanib	expenditure cap	1-Nov-2015	Under EEE	
Naglazyme	galsulfase	refund	1-Mar-2016	refund pilot program (1-Oct-2012), Essential drug	
Stivarga	regorafenib	refund	1-Jun-2016		
Vimizim	elosulfase alfa	expenditure cap	1-Jun-2016	Under EEE	
Pomalyst	pomalidomide	refund	1-Jan-2017		
Defitelio	defibrotide	expenditure cap	1-Jun-2017	Under EEE	
Perjeta	pertuzumab	time cap per patient	1-Jun-2017		
Zelboraf	vemurafenib	expenditure cap	1-Jul-2017	Under EEE	
Kadcyla	trastuzumab emtansine	utilization cap	3-Aug-2017		
Opdivo	nivolumab	refund + expenditure cap	21-Aug-2017		
Keytruda	pembrolizumab	refund + expenditure cap	21-Aug-2017		
Rafinlar	dabrafenib	expenditure cap	1-Sep-2017	Under EEE	
Lynparza	olaparib	expenditure cap	1-Oct-2017	Under EEE	
Meqsel	trametinib	expenditure cap	1-Nov-2017	Under EEE	
Ibrance	palbociclib	refund	6-Nov-2017		
Tagrisso	osimertinib	discounted treatment initiation	5-Dec-2017		
Tecentriq	atezolizumab	expenditure cap	12-Jan-2018	Under EEE	
Sylvant	siltuximab	expenditure cap	1-Feb-2018	Under EEE	
Kyprolis	carfilzomib	refund	5-Feb-2018		
Iclusig	ponatinib	expenditure cap	1-Apr-2018	Under EEE	
Imbruvica	ibrutinib	expenditure cap	1-Apr-2018	Under EEE	
Cyramza	ramucirumab	refund	1-May-2018		
Blincyto	blinatumomab	expenditure cap	1-Jul-2018	Under EEE	
Vyndaqel	tafamidis	expenditure cap	1-Oct-2018	Under EEE	
Cabometyx	cabozantinib	refund + expenditure cap	1-Feb-2019		
Praxbind	idarucizumab	expenditure cap	1-Feb-2019	Under EEE	
Darzalex	daratumumab	refund + expenditure cap	8-Apr-2019		
Spinraza	nusinersen	refund + expenditure cap	8-Apr-2019		
*Limited activation*					
Olita	olmutinib	expenditure cap	15-Nov-2017	Under EEE	
Lartruvo	olaratumab	expenditure cap	1-Dec-2018	Under EEE	
*Expired activation*					
Evoltra	clofarabine	CED	11-Dec-2013 (1-Dec-2018)		the first RSA drug, conventional reimbursement after re-evaluation
Revlimid	lenalidomide	refund	5-Mar-2014 (1-Dec-2017)		conventional reimbursement due to generic
Xalkori	crizotinib	refund	1-May-2015 (1-May-2019)		conventional reimbursement due to alternative drugs after re-evaluation
Pirespa	pirfenidone	refund	3-Oct-2015 (1-Nov-2017)		conventional reimbursement due to generic
Diterin	sapropterin	expenditure cap	1-Nov-2017 (8-May-2019)		Under EEE, conventional reimbursement due to generic
Alecensa	alectinib hydrochloride	expenditure cap	1-Oct-2017 (1-Dec-2018)		Under EEE, conventional reimbursement due to alternative drugs after expansion of the indication

*RSA* risk sharing arrangement, *EEE* exemption of economic evaluation, *CED* coverage with evidence development

Eculizumab and galsulfase were first reimbursed by the refund pilot program, which was launched in August 2009. This also enabled the NHIS to receive “pay-back” from pharmaceutical companies with a certain fraction of the drug price. Although it was initially intended to last only 1 year, several extensions took place until RSAs were introduced. Following the termination of the pilot program, the DREC appraised their eligibility for RSAs.

Two drugs had limited activation (olmutinib and olaratumab). The MFDS had granted them conditional approval; however, their phase III clinical trials failed and the manufacturers abandoned their development. Therefore, these two drugs are only available for current patients who have been treated with them (i.e., dispensing of these medications for new patients is not allowed).

Four drugs were re-evaluated before the termination of their 4-year activation period: cetuximab, enzalutamide, clofarabine, and crizotinib. The first two were successful at obtaining re-agreements with the same type. Although the other two drugs were unsuccessful, the DREC confirmed their reimbursement through conventional appraisal– the clinical efficacy of clofarabine was proven and alternative agents to crizotinib were revealed. Accordingly, these two drugs are no longer under RSAs; however, they are reimbursed in a similar way to non-RSA drugs. Meanwhile, the activation of three drugs (pirfenidone, lenalidomide, and sapropterin) was terminated due to the listing of generics before the end of the contracted period. One drug (alectinib) was converted to conventional reimbursement from RSAs because it added a new indication for which there were alternative drugs.

Table 3 shows that access to RSA drug has accelerated in South Korea. On average, approximately 850 days had passed for all RSA drugs before the start of reimbursement following market approval. They were divided into three groups according to the MFDS approval year based on time of policy introduction – 2013 for RSAs and 2015 for EEE.

**Table 3:** Days between approval and reimbursement and the counts of appraisal for reimbursement of RSA drugs in South Korea

(unit: day, count)				
		Total	RSA only	RSA with EEE
All drugs	N	39	21	18
	days between approval and reimbursement	849.4	912.1	776.3
	average number of appraisals^b^	1.92	2.24	1.56
Market approval by MFDS before 2014	N	12	10	2
	days between approval and reimbursement	1167.4	1131.4	1347.5
	average number of appraisals^b^	2.83	2.70	3.50
Market approval by MFDS in 2014-2015	N	14	5	9
	days between approval and reimbursement	923.7	1010.0	875.8
	average number of appraisals^b^	1.64	1.80	1.56
Market approval by MFDS after 2015^a^	N	13	6	7
	days between approval and reimbursement	475.9	465.0	485.3
	average number of appraisals^b^	1.38	1.83	1.00

The activation date of eculizumab and galsulfase was applied with the refund pilot program (October 1, 2012 and December 1, 2009, respectively)

^a^until the first half of 2019

^b^not including deferred appraisal

*MFDS* Ministry of Food and Drug Safety, *RSA *risk sharing arrangement, *EEE *exemption of economic evaluation

A third of RSA drugs were approved after 2016. The time gap between market approval and reimbursement was remarkably decreased. Drugs approved before RSAs took 1,167 days for reimbursement. After the introduction of RSAs, 924 days were taken, a decrease of eight months. Finally, it was reduced by almost 60% — 476 days after the introduction of EEE. A similar trend was found for the average number of appraisals by the DREC for each drug (approximately 2.8 number of times for drugs approved before 2014 and 1.4 for drugs approved after 2016).

Kim analyzed the impact of some policies on drug reimbursement, including RSAs and EEE [36]. After the introduction of a positive listing system, 430 new drugs were appraised by the DREC between January 2007 and May 2018. Among them, cancer drugs and orphan drugs for rare diseases accounted for 30.2% (130 drugs). The policies of RSAs and EEE dramatically increased the rate of DREC approval for cancer and orphan drugs. It was only 40.8% between 2007 and 2013; but increased to 50.0% after the introduction of RSAs; and doubled after the introduction of EEE (82.0%). In the case of other new drugs, these percentages were 68.4%, 79.3%, and 80.8%, respectively.

We carried out a comparative analysis of the risk sharing and other policies on patients’ access in South Korea, NHS England, Italian SSN, and Australian PBS. Based on our findings, there were considerable overlaps between these three countries and South Korea. Like in South Korea, financial-based schemes were a common type of risk sharing in the UK and Australia. Table 4 shows types of risk sharing and their number of applied indications. There were 64 indications for 39 Korean RSA drugs. In the case of Korean NHI, 52 indications (81.3%) were reimbursed. The remaining 12 indications were not reimbursed or approved in South Korea. In the UK, 38 indications (59.4%) were reimbursed. Among them, 33 indications were under PAS – 27 for simple discount and 5 for complex scheme. Further, there was a combination of PAS and CDF (daratumumab). Fourteen indications (21.9%) were subsidized by CDF. In Italy, 59 indications (92.2%) were reimbursed and nine of them had MEAs – five for outcome-based and four for financial-based. A total of 39 indications (60.9%) were reimbursed by the Australian PBS. Mixed schemes were very common in Australia. MES was only applied for 4 indications in combination with other access schemes. 32 indications managed their financial risks by RSAs and/or SPAs. Trametinib was the only drug that applied those three arrangements all together. Finally, three drugs were listed in the LSDP. The supplementary table shows more details about this comparison.

**Table 4:** Comparison of Korean RSA drugs with the UK, Italy, and Australia

	No. of indications	% of reimbursed
Total	64	100.0
South Korea		
RSA		
refund	11	
expenditure cap	22	
refund + expenditure cap	7	
others	3	
conventional reimbursement	9	
*sub total*	52	81.3
not reimbursed	11	
not approved	1	
UK		
PAS		
simple discount	27	
complex scheme	5	
PAS + CAA	1	
conventional reimbursement	5	
*sub total*	38	59.4
CDF	14	21.9
not reimbursed	12	
Italy		
MEA		
financial-based	4	
outcome-based	5	
conventional reimbursement	50	
*sub total*	59	92.2
not reimbursed	5	
Australia		
MES	0	
financial RSA	2	
SPA	4	
financial RSA + SPA	26	
MES + SPA	1	
MES + financial RSA	2	
MES + financial RSA + SPA	1	
conventional reimbursement	3	
*sub total*	39	60.9
LSDP	3	4.7
not reimbursed	22	

*RSA* risk sharing arrangement, *PAS* patients access scheme, *CAA* CDF (cancer drug fund) managed access agreement, *CDF* cancer drug fund, *MEA* managed entry agreement, *MES* managed entry scheme, *SPA* special pricing arrangements, *LSDP* life-saving drugs program

## Discussion

There are some uncertainties regarding pharmaceutical reimbursement based on clinical evidence and cost-effectiveness. RSAs are mechanisms that share these risks between stakeholders. In 2013, the Korean government introduced RSAs as part of the NHI coverage expansion plan for cancer drugs and orphan drugs. Additionally, there is the EEE policy for the waiver of economic evaluation introduced in 2015. In South Korea, 39 drugs took advantage of RSAs – 31 had effective activation, 2 were only limited to pre-treated patients, and the activation of 6 was expired; however, they were still reimbursed in a conventional manner. Almost all were arranged by a financial-based scheme. Refund was identified as the majority type and all expenditure caps were applied for drugs under EEE. The time gap between market approval by the MFDS and reimbursement by the NHIS for RSA drugs was decreased. Additionally, a decrease in the number of DREC appraisal per RSA drug was observed. This helped to shorten the time gap and consequently alleviate patients’ financial burden by virtue of subsidy by NHI. To compare 39 RSA drugs in South Korea and their various indications, we examined the drug lists in the UK, Italy, and Australia. The types of risk sharing varied across countries and the indications under RSAs differed according to each jurisdiction despite being from the same drug. In Italy, more than 90% of indications were included; this was followed by South Korea (81.3%), Australia (60.9%), and the UK (51.6%). But including CDF, the proportion of indications being subsidized by government is almost three-fourths in the UK. This means CDF plays a significant role in patients' access to drugs in the UK. In any case, it can be said that Korean patients’ access to these new drugs is not limited relative to these countries.

In most countries, health authorities have attempted to find a balance between cost containment and the expansion of coverage. Notably, the improvement of patients’ early access to new drugs through RSAs has been highlighted [2–6, 15, 37–39]. Similarly, we found some significant results from the experiences of South Korea. Before RSAs were introduced, new drugs that had uncertainties regarding their cost-effectiveness tended to be rejected or were assigned a pending status by the DREC. Once a decision was deferred, it was inevitable to prepare for the next meeting. Pharmaceutical companies were asked to modify their rejected strategies (e.g., drug price and model of economic evaluation) and re-submit their applications for reimbursement. Accordingly, the number of DREC’s appraisals and the time to receiving a reimbursement were increased inevitably. In fact, these drugs may have not been reimbursed if RSAs had not been introduced. The criteria of being eligible for RSAs could help the DREC to arrive at a decision more quickly. Consequently, less than two rounds of appraisal meetings were required to make a positive decision after 2013. Therefore, RSAs and EEE in South Korea could be evaluated as a contribution to patients’ access to medicine.

RSA also led to the alleviation of patients’ out-of-pocket expenses for new drugs. According to the HIRA’s report, patients’ out-of-pocket expenses were annually reduced by KRW 130 billion (USD 150.7 million using purchasing power parity in 2016) based on a scenario analysis for the early adopted 11 RSA drugs [40]. All stakeholders, including patient group, policymakers, healthcare professionals, non-governmental organizations, and pharmaceutical companies, appreciated the value of Korean RSAs because of their access to medicine [40].

The coverage level of Korean RSAs was not found to be insufficient compared to that of the UK, Australia, and Italy. However, the Korean RSAs only apply to cancer and rare diseases while in the other three countries, there is no restriction for diseases. Nevertheless, cancer drugs account for most of the RSA drugs in these countries. This is because they are lifesaving and very expensive [5, 41]. There seems to be more focus on financial-based schemes in all four countries. However, this tendency could be easily found in other countries. The development and management of RSAs result in administrational and bureaucratic burden. Further, transaction time and the costs for executing RSAs to all stakeholders are burdensome with performance-based schemes being more difficult than financial-based schemes [2, 5, 41]. To adopt performance-based schemes, payers should be prepared to measure appropriate health outcomes. The paucity of guidelines on performance-based schemes might encourage the development of the financial-based scheme. In terms of complexity, preference for financial-based schemes is somewhat of a natural consequence. However, there are differences in the implementation of RSAs between countries just as the names of policy differ between countries. These diversities are derived from the circumstances of each country, including public demands, health system, eligibility criteria for risk sharing, and evaluation framework for reimbursement.

In South Korea, RSAs provide a non-conventional framework for covering new drugs under NHI. The criterion, “no alternative treatment,” plays a very important role at the initial evaluation and the re-evaluation of RSA drugs. The RSA contracts for five drugs were terminated because they did not satisfy the criterion; three were faced with their generics during their contract period while the other two had therapeutically equivalent drugs available at the time of re-evaluation. “No alternative treatment” is a very important criterion that is used by the DREC during their decision-making regarding reimbursement.

In South Korea, the issue of type of diseases that are eligible for RSAs was raised. Specific protocols have been derived to implement RSAs for cancer and orphan drugs alone. Recently, the MoHW added detailed requirements for the regulation of RSAs with drugs for other diseases; (1) drugs without alternative therapeutic treatments, (2) drugs for severe incurable diseases, (3) drugs that offer clinically significant improvement to patients’ quality of life, and (4) drugs approved as breakthrough therapy designation (BTD) or priority medicines (PRIME) by the US Food and Drug Administration (FDA) or the European Medicines Agency (EMA). Drugs that satisfy the four aforementioned conditions could be reimbursed with RSAs. Since 2020, dupilumab for moderate-to-severe atopic dermatitis has been reimbursed with RSAs. In fact, it is the first non-cancer, non-orphan drug reimbursed with RSAs in South Korea. Despite the extension of the therapeutic area, “no alternative” is still emphasized as a strict rule for evaluation.

Like other policies, RSAs have both advantages and disadvantages. Although policymakers implementing RSAs could lessen the uncertainty in cost-effectiveness and clinical benefit, there remains some budgetary matters (i.e., as many patients get early access, a greater budget pressure would ensue). A lack of transparency also serves as another issue [2, 5, 37, 42]. In most cases, the detailed condition of agreements and the results from evaluating patients’ health outcome remain confidential. Refund is a simple and widespread type of risk sharing; however, it is often criticized because of its dual price system. Regarding the performance-based scheme, if data were unveiled, additional efforts to generate clinical evidence could be avoided. Although pharmaceutical companies may not be comfortable with data release, it is imperative that they bear in mind that patients and payers have been willing to share their information to aid in the generation of evidence for the new drugs.

This study had some limitations. First, we could only access and collect data published online in Korean or English. Because almost all conditions surrounding the agreements were confidential in South Korea, we could not estimate the actual expenditure of NHI nor the reduction of patients’ out-of-pocket payments due to RSAs. Further, confidentiality has been observed in the other three countries. In Australia, a final decision on the PBAC’s recommendation was not disclosed. Thus, of the 39 RSA drugs, we only examined the PBAC’s outcomes for MEAs/RSAs of the drugs listed in the pharmaceutical schedules.

## Conclusion

This study showed that RSAs in South Korea improved patients' access to new drugs to a certain extent. Further, it revealed that drug coverage of Korean NHI had a level comparable to that of other countries. Our findings could thus serve as reference points for countries that have a dual mission of containing pharmaceutical expenditure and improving access to new drugs.

## Supplementary Information

**Additional file 1: Supplementary table. **RSA drugs and the indication they are administered to treat in South Korea compared to the UK, Italy, and Australia.

## Data Availability

The datasets generated and/or analyzed during the current study are available as follows - Korean data: Drugs Reimbursement Evaluation Committee final recommendation in the HIRA website, http://www.hira.or.kr/bbsDummy.do?pgmid=HIRAA030014040000 [22]. List of reimbursed drugs and prices in the HIRA website, http://www.hira.or.kr/bbsDummy.do?pgmid=HIRAA030014050000 [23]. Information of drug approval in the MFDS website. https://nedrug.mfds.go.kr/searchDrug [24]. - the UK data: NHS England Drugs List in NHS England website, https://www.england.nhs.uk/wp-content/uploads/2019/04/nhs-england-drugs-list-v14.1.pdf [26]. PAS list in the NICE website, https://www.nice.org.uk/Media/Default/About/what-we-do/PASLU/NICE-recommended-technologies-that-include-a-commercial-arrangement.xlsx [27]. CDF list in NHS England website, https://www.england.nhs.uk/wp-content/uploads/2017/04/national-cdf-list-v1.135.pdf [28]. - Italian data: Drug list on class A and class H in AIFA website, http://www.agenziafarmaco.gov.it/content/elenco-medicinali-di-fascia-e-h [29]. MEA list in AIFA website, http://www.agenziafarmaco.gov.it/content/comunicazioni-managed-entry-agreements-mea [30]. - Australian data: General Pharmaceutical Schedule in the PBS website, https://www.pbs.gov.au/publication/schedule/2019/06/2019-06-01-general-schedule-volume-1.pdf [33]. Pharmaceutical Schedule - Sect. 100 Schedule in the PBS website, https://www.pbs.gov.au/publication/schedule/2019/07/2019-07-01-section100-volume-2.pdf [34]. Pharmaceutical Schedule - Efficient Funding of Chemotherapy in the PBS website, https://www.pbs.gov.au/publication/schedule/2019/07/2019-07-01-efc.pdf [35]. List of LSDP in the PBS website, https://www.health.gov.au/internet/main/publishing.nsf/Content/lsdp-criteria [20].

## Abbreviations

AIFA: Agenzia Italiana del Farmaco
CDF: Cancer drugs fund
DoH: Department of Health
DREC: Drug Reimbursement Evaluation Committee
EEE: Exemption of economic evaluation
HIRA: Health Insurance Review and Assessment Service
LSDP: Life saving drugs program
MEA: Managed entry agreement
MES: Managed entry schemes
MFDS: Ministry of Food and Drug Safety
MoHW: Ministry of Health and Welfare
NHI: National health insurance
NHIS: National Health Insurance Service
NHS: National health service
NICE: National Institute for Health and Care Excellence
PAS: Patient access schemes
PBAC: Pharmaceutical Benefits Advisory Committee
PBS: Pharmaceutical Benefits Scheme
RSA: Risk sharing arrangement
SPA: Special pricing arrangement
