# Supplementary material for: How can we improve patients’ access to new drugs under uncertainties? : South Korea’s experience with risk sharing arrangements
Source: BMC Health Serv Res. 2021 Sep 14;21:967. doi: 10.1186/s12913-021-06919-x (PMC8442279; doi:10.1186/s12913-021-06919-x)
Supplement: Supplementary file 1 — Additional file 1: Supplementary table. RSA drugs and the indication they are administered to treat in South Korea compared to the UK, Italy, and Australia. [file 12913_2021_6919_MOESM1_ESM.docx]

**Supplementary table.** RSA drugs and the indication they are administered to treat in South Korea compared to the UK, Italy, and Australia

| Active substance | Indication | South Korea | UK | Italy | Australia |
| --- | --- | --- | --- | --- | --- |
| cetuximab | colorectal cancer | refund | simple discount | conventional | SPA |
|  | squamous-cell carcinoma of head and neck | conventional  (100/100) | CDF | conventional | financial-RSA (rebate+subsidization cap) |
| enzalutamide | castration-resistant prostate cancer | refund | simple discount | conventional | SPA + financial RSA  (subsidization cap) |
| eculizumab | paroxysmal nocturnal hemoglobinuria | refund | conventional | conventional | LSDP |
|  | atypical haemolytic uraemic syndrome | refund | conventional | conventional | MES + financial RSA (hard cap) |
| vandetanib | metastatic medullary thyroid cancer | expenditure cap | X | financial-based | X |
| galsulfase | MPS^§^ VI (Maroteaux-Lamy syndrome) | refund | conventional | conventional | LSDP |
| regorafenib | gastrointestinal stromal tumor | refund | simple discount | conventional | X |
|  | hepatocellular carcinoma | refund | simple discount | conventional | X |
|  | metastatic colorectal cancer | X | X | conventional | X |
| elosulfase alfa | MPS^§^ VIa (morquio A syndrome) | expenditure cap | simple discount | conventional | LSDP |
| pomalidomide | multiple myeloma | refund | simple discount | conventional | SPA + financial RSA  (patient number cap) |
| defibrotide | hepatic veno-occlusive disease after HSCT | expenditure cap | conventional | X | X |
| pertuzumab | HER2 positive breast cancer | time cap  per patient | CDF | conventional | financial-RSA |
|  | Neoadjuvant treatment of breast cancer | X | simple discount | conventional | X |
| vemurafenib | metastatic melanoma | expenditure cap | simple discount | outcome-based | SPA +  financial RSA (cap) |
| trastuzumab emtansine | HER2 positive breast cancer | utilization cap | simple discount | outcome-based | conventional |
| nivolumab | non-small cell lung cancer | refund + expenditure cap | CDF | conventional | SPA +  financial RSA  (subsidization cap) |
|  | metastatic melanoma | refund + expenditure cap | CDF | conventional | SPA +  financial RSA  (subsidization cap) |
|  | renal cell carcinoma | X | simple discount | conventional | SPA +  financial RSA  (subsidization cap) |
|  | Hodgkin lymphoma | X | simple discount | conventional | X |
|  | squamous-cell carcinoma of head and neck | X | CDF | conventional | SPA +  financial RSA  (subsidization cap) |
|  | urothelial carcinoma | X | X | conventional | X |
| pembrolizumab | non-small cell lung cancer | refund + expenditure cap | CDF | conventional | SPA +  financial RSA  (subsidization cap) |
|  | metastatic melanoma | refund + expenditure cap | CDF | conventional | MES + financial RSA  (annual expenditure cap) |
|  | urothelial carcinoma | X | CDF | conventional | SPA + financial RSA  (expenditure cap) |
|  | Hodgkin lymphoma | X | CDF | conventional | SPA +  financial RSA  (subsidization cap) |
|  | squamous cell carcinoma of the head and neck | X | X | conventional | X |
| dabrafenib | metastatic melanoma | expenditure cap | simple discount | outcome-based | SPA +  financial RSA (cap) |
| olaparib | BRCA mutation-positive ovarian cancer | expenditure cap | time cap | conventional | SPA +  financial RSA  (expenditure cap) |
|  | fallopian tube cancer | expenditure cap | time cap | conventional | SPA +  financial RSA  (expenditure cap) |
|  | primary peritoneal cancer | expenditure cap | time cap | conventional | SPA +  financial RSA  (expenditure cap) |
| trametinib | metastatic melanoma | expenditure cap | simple discount | conventional | MES + SPA + financial RSA |
| palbociclib | HER2 negative breast cancer | refund | simple discount | conventional | X |
|  | metastatic breast cancer | X | X | X | SPA +  financial RSA  (subsidization cap) |
| osimertinib | non-small cell lung cancer | discounted treatment initiation | CDF | conventional | SPA +  financial RSA  (expenditure cap) |
| atezolizumab | non-small cell lung cancer | expenditure cap | simple discount | conventional | SPA +  financial RSA  (subsidization cap) |
|  | urothelial carcinoma | expenditure cap | simple discount | conventional | X |
| siltuximab | multicentric castleman‘s disease | expenditure cap | X | conventional | X |
| carfilzomib | multiple myeloma | refund | simple discount | financial-based | SPA |
| ponatinib | acute lymphoblastic leukemia | expenditure cap | simple discount | conventional | conventional |
|  | chronic myeloid leukemia | expenditure cap | simple discount | outcome-based | conventional |
| ibrutinib | chronic lymphatic leukemia | expenditure cap | simple discount | conventional | SPA +  financial RSA  (expenditure cap) |
|  | mantle cell lymphoma | expenditure cap | CDF | conventional | SPA +  financial RSA  (subsidization cap) |
|  | Waldenstroms macro-globulinaemia | expenditure cap | CDF | conventional | X |
| ramucirumab | gastric cancer or gastro–oesophageal junction adenocarcinoma | refund | X | financial-based | X |
| blinatumomab | acute lymphoblastic leukemia | expenditure cap | simple discount | outcome-based | SPA +  financial RSA (financial cap) |
| tafamidis | transthyretin familial amyloid polyneuropathy | expenditure cap | X | conventional | X |
| cabozantinib | clear-cell type renal cancer | refund + expenditure cap | simple discount | conventional | SPA +  financial RSA (cap) |
|  | medullary thyroid cancer | X (not approved) | simple discount | X | X |
| idarucizumab | acute reversal agent for anticoagulation† | expenditure cap | conventional | conventional | X |
| daratumumab | multiple myeloma | refund + expenditure cap | PAS + CAA | conventional | X |
| nusinersen | 5q spinal muscular atrophy | refund + expenditure cap | X | conventional | SPA +  financial RSA |
| olmutinib | non-small cell lung cancer | expenditure cap | X | X | X |
| olaratumab | soft tissue sarcoma | expenditure cap | CDF^*^ | conventional^*^ | X |
| clofarabine | acute lymphoblastic leukemia^‡^ | conventional | CDF | X | X |
| lenalidomide | multiple myeloma | conventional | dose cap | conventional | SPA +  financial RSA (cap) |
|  | myelodysplastic syndromes^⁋^ | conventional | dose cap | conventional | SPA +  financial RSA  (additional cost) |
|  | mantle cell lymphoma | X | X | conventional | X |
| crizotinib | ALK positive non-small cell lung cancer | conventional | simple discount | conventional | MES + SPA |
|  | ROS1-positive advanced non-small-cell lung cancer | conventional | simple discount | conventional | SPA |
| pirfenidone | idiopathic pulmonary fibrosis | conventional | simple discount | conventional | SPA +  financial RSA (cap) |
| sapropterin | phenylketonuria | conventional | X | conventional | SPA +  financial RSA  (expenditure cap) |
| alectinib | non-small cell lung cancer | conventional | simple discount | financial-based | SPA |

^§^mucopolysaccharidosis; ^†^specific reversal agent for dabigatran; ^‡^under the age of 21 years; ^⁋^associated with an isolated deletion 5q cytogenetic abnormality, ^*^European Medicines Agency (EMA) recommends the withdrawal of marketing authorization for the cancer medicine, olaratumab (Lartruvo), in January 2019.
In the case of Korea, “not approved” indication is not yet approved by the MFDS; thus, any commercial approach is not permitted.
RSA, risk sharing arrangement; PAS, patients access scheme; MEA, managed entry agreement; CDF, cancer drug fund; LSDP, life-saving drugs program; CAA, CDF (cancer drug fund) managed access agreement; SPA, special pricing arrangements; MES, managed entry scheme; HER2, human epidermal growth factor receptor type 2; BRCA, breast cancer susceptibility gene; ALK, anaplastic lymphoma kinase; ROS1, ROS (reactive oxygen species) proto-oncogene 1
